# Factors Affecting the Quality of Work Life of Nurses at Tertiary General Hospitals in the Context of the COVID-19 Pandemic

**DOI:** 10.3390/ijerph19084718

**Published:** 2022-04-13

**Authors:** Eunhee Hwang

**Affiliations:** Department of Nursing, Wonkwang University, Iksan 54538, Korea; ehh@wku.ac.kr; Tel.: +82-63-850-6071

**Keywords:** job stress, mindfulness, registered nurse, quality of life

## Abstract

The prolonged coronavirus disease 2019 (COVID-19) pandemic has caused an overload of work for nurses and resulted in high levels of stress. Improving the quality of work life may be a useful mediator for these demands. The purpose of this study was to identify factors affecting work stress, turnover intention, mindfulness, and quality of work life in nurses working in tertiary general hospitals. The participants of this study were 207 female nurses working in tertiary general hospitals with more than six months of clinical experience. Data were collected using an online Google survey. Data were analyzed using the SPSS/WIN 26.0 program. The mean score for the quality of work life was 3.81 ± 0.53 out of six points. The quality of work life was negatively correlated with job stress (r = −0.36, *p* < 0.001) and turnover intention (r = −0.45, *p* < 0.001) and positively correlated with mindfulness (r = 0.35, *p* < 0.001). Factors affecting quality of work life were work satisfaction (β = 0.27, *p* = 0.004), job stress (β = −0.23, *p* < 0.001), and turnover intention (β = −0.18, *p* = 0.016). As a result, positive factors such as work satisfaction had stronger effects than negative factors. Thus, it would be necessary to seek strategies such as improving compensation for nurses, enhancing teamwork, or establishing a support system for managers, superiors, and colleagues.

## 1. Introduction

Recently, nurses in Korea are facing various concerns such as bullying in the workplace, suicide, and frequent turnover [1,2]. This has led to increased demand for improved work environment and efficient manpower management of nurses. In addition, the prolonged coronavirus disease 2019 (COVID-19) pandemic has caused an overload of work for nurses and resulted in high levels of stress [3]. In the initial days of the pandemic, nurses provided the best care for patients with a sense of duty and sacrifice in accordance with the quarantine guidelines from the government. However, the pandemic, which has lasted for over two years, has increased the demand for an improved work environment [4]. In addition, there is a relative lack of interest in the individual needs and quality of life of nurses, due to the common belief that nurses must always prioritize patient care over personal life. Improving the quality of work life, which has recently become an issue, may be a useful response to these demands.

The quality of work life is the level of subjective satisfaction at work while achieving organizational goals [5]. Following rapid economic growth, the interest in well-being and pursuing happiness in life has increased. As a result, the workplace is recognized as a place for self-actualization that enables individual satisfaction and growth rather than simply serving an environment in which achieve economic aims [6]. The quality of work life is not only related to work, but also to how organizations enable individual holistic well-being [7].

The literature has pointed out important work attitudes and behaviors that may be affected by quality of work life, such as vigor, dedication, motivation, commitment, adaptability to changes in the work environment, creativity, desire to innovate and even intent to stay or leave the organization/profession [8,9,10]. In hospitals with a low quality of work life, the absence and turnover rates for nurses were higher than the average, and improvements in the quality of work life led to an increased work performance and reduced burnout, absence, turnover rate, and stress [5,11]. Based on the above discussion, it is assumed that an improvement in work life quality may be the solution to various problems regarding nurses and the work environment.

Job stress is a harmful physical and mental response observed when work demands do not match the individual’s resources, capabilities, and needs, and is closely related to the organizational structure, characteristics and environment of the job [12,13]. During the COVID-19 pandemic, clinical nurses experienced increased work stress, not only due to the special circumstances of the pandemic, but also due to their additional duties and burdens. Clinical nurses showed high work stress during the pandemic, and this high level of stress had the greatest effect on nurse burnout [3]. Moreover, nurses’ work stress affected the quality of their work life [14].

Turnover intention is the intent of an employee to leave an organization in search of a new job [15]. Turnover intention leads to change in occupation and affects the quality of work life [16]. Azevedo, Nery, and Cardoso [17] pointed to the following characteristics of nurses with a low quality of work life: turnover consideration, less than seven hours of sleep, low support from colleagues or superiors, and working in the intensive care unit (ICU). Nurse turnover has a negative impact on the ability to understand patient needs and deliver high standards of care, and leads to insufficient staffing, which increases the workloads and stress on other staff [18,19,20,21]. However, other studies reported conflicting findings in which there was no correlation between turnover intension and quality of work life [22]. Thus, further studies should investigate these variables.

Mindfulness improves attention and awareness of present experiences or existing realities. Mindfulness allows one to focus and become more aware of current emotions, and thus, one is less likely to perceive stress and shows improved coping with stress [23]. In fact, mindfulness alleviates negative factors such as stress, burnout, depression, and anxiety and enhances positive factors, including happiness, empathy, and life satisfaction [24,25]. During the COVID-19 pandemic, it may be meaningful to determine whether mindfulness can positively affect quality of work life in nurses, for future policy establishment.

In a previous study on the quality of work life of nurses in Brazil, 36% of nurses were not satisfied with their quality of work life [17]. As previously described, quality of work life not only affects organizational performance, but also individual life. Moreover, patient outcomes are influenced by clinical treatments and interventions as well as work–life balance and the quality of life of health managers [26,27]. As the COVID-19 pandemic continues, it would be timely and adequate to investigate the quality of work life in nurses.

Therefore, the purpose of this study was to identify the factors affecting quality of work life in nurses working in tertiary general hospitals. The specific aims of this study were as follows:

1. To assess work stress, turnover intention, mindfulness, and quality of work life in nurses;
2. To investigate differences in the quality of work life according to the demographic features of nurses;
3. To analyze the correlation between work stress, turnover intention, mindfulness, and quality of work life;
4. To identify the factors that affect the quality of work life.

## 2. Materials and Methods

### 2.1. Design

This study is a descriptive study to identify the factors affecting the quality of work life in nurses working in tertiary general hospitals. 

### 2.2. Participants

The participants of this study were female nurses working in tertiary general hospitals with more than six months of clinical experience. In Korea, although the participation rate of women in economic activities is increasing, the traditional gender roles regarding the division of labor remain to some extent; social support to help maintain a work–family balance is increasing, but is still in an insufficient, transitional state [28]. These cultural characteristics can explain some of the gender differences in factors affecting quality of life [29]. In addition, based on previous studies [30,31] that reported gender differences in the quality of work life, this study selected female nurses, who make up most nurses, in order to exclude such gender differences. A regression analysis was conducted using G*power program 3.1.9.2 with a significance level of 0.05, median effect size of 0.15, power of 0.9, and 15 predicting variables to calculate the minimum number of participants that were required for this study. A minimum of 199 participants was required. Considering possible withdrawals and dropouts, a total of 223 participants were enrolled in this study to complete the questionnaires. A total of 207 questionnaires were analyzed, excluding 16 questionnaires with incomplete responses.

### 2.3. Measurements

#### 2.3.1. Quality of Work Life

Quality of work life was evaluated using Quality of Nursing Work life, developed by Brooks and Anderson [5], and translated into Korean and modified by Kim and Ryu [6]. The tool consisted of five sub-domains: hospital management (10 items), communication and teamwork (seven items, hospital welfare policies (seven items), work design for quality nursing (10 items), and working conditions (seven items). This self-administered questionnaire included 41 items, which were evaluated on a six-point Likert scale, from one point for ‘strongly disagree’ to six points for ‘strongly agree’. A higher scored indicated a higher quality of work life. Cronbach’s α of the tool was 0.92 in the study by Kim and Ryu [6] and 0.90 in this study.

#### 2.3.2. Job Stress

Job stress was evaluated using a tool developed by Ku and Kim [32], which was verified for construct validity by Joo [33]. The tool consisted of 43 items in nine sub-domains: nursing work (six items), role conflict (five items), professional knowledge (four items), conflict with physicians (three items), psychological burden (three items), interpersonal relationship (six items), nurse compensation (five items), work schedule (seven items), and patients and caregivers (four items). The items were evaluated on a five-point Likert scale, and a higher score indicated greater work stress. Cronbach’s α of the tool was 0.82 in a study by Lee et al. [34] and 0.94 in this study.

#### 2.3.3. Turnover Intention

Turnover intention was evaluated using a tool developed by Becker [35]. The tool was later modified by Kim [36] for word choices that were more suitable for hospital environment and nurses. A total of six items was included in the tool, and the items were evaluated on a five-point Likert scale, from one point for ‘strongly disagree’ to five points for ‘strongly agree’. Item four was reverse-coded. A higher score indicated greater turnover intention. Cronbach’s α was 0.89 in a study by Park [37] and 0.85 in this study.

#### 2.3.4. Mindfulness

Mindfulness was evaluated using a mindfulness scale developed by Park [38] The tool consisted of four sub-domains: awareness of present time, attention, non-judgmental acceptance, and decentralized attention. Each domain contained five items, and each item was evaluated on five-point Likert scale, from one point for ‘strongly disagree’ to five points for ‘strongly agree’. The items were reverse-scored, and a higher total score indicated higher level of mindfulness. Cronbach’s α of the tool was 0.88 at the time of development by Park [38] and 0.93 in this study.

### 2.4. Data Collection

After approval by the Institutional Review Board (IRB), data were collected from 17 January to 5 February 2022. The purpose and method of the study, anonymity, and confidentiality were explained to the head of the institution. Data were collected after obtaining permission. Data were collected using an online Google survey. The Google Link survey included a consent form with explanations of the study purpose, the collection of personal information, and confidentiality on the first page. Thus, only those participants who voluntarily agreed to participate were enrolled. In addition, the responses were automatically processed after completion of the questionnaire through a computerized system, and the responses could not be used to identify the participants.

### 2.5. Data Analysis 

The collected data were analyzed using the SPSS/WIN 26.0 software. Descriptive statistics were analyzed for general characteristics, work stress, turnover intention, mindful-ness, and quality of work life. A *t*-test, ANOVA, and Tukey post-hoc test were conducted to compare differences in the quality of work life according to general characteristics. Pearson’s correlation analysis was conducted to analyze the correlation between work stress, turnover intention, mindfulness, and quality of work life. Multiple regression analysis was performed to identify the factors affecting the quality of work life. 

## 3. Results

### 3.1. Demographic Features

A total of 207 participants with a mean age of 36.11 ± 9.16 were included in this study. Among them 98 (47.3%) and 107 (52.7%) were single and married, respectively. For education level, 139 (67.1%) participants had a bachelor’s degree. A total of 73 (35.3%) participants had a total household income of greater than USD 5000, and 62 (30.0%) participants had a total household income between USD 2500 and 3300. Most participants (159 participants, 76.8%) were staff nurses, and 106 (51.2%) and 101 (48.8%) were full-time and shift workers, respectively. In addition, 68 (32.9%) participants worked in the general ward, followed by 61 (29.5%) in the outpatient department, 29 (14.0%) in other departments, 27 (13.0%) in special departments (emergency room, operating room, recovery room, delivery room, neonatal room), and 22 (10.6%) in the ICU. The mean total clinical experience of the participants was 157.34 ± 111.34 months, and the mean clinical experience in the current department was 54.73 ± 60.49 months. To increase their satisfaction with the current work, the greatest number of participants (98 participants, 47.3%) felt moderately satisfied, and 73 (35.3%) answered that their health condition was moderate. A total of 80 (38.6%) participants had regular eating habits, and 47 (22.7%) participants exercised regularly (Table 1).

### 3.2. Job Stress, Turnover Intention, Mindfulness, and Quality of Work Life of Participants

The mean score for work stress was 4.17 ± 0.42 out of five points. In more detail, among the sub-domains, conflict with physicians had the highest score, at 4.54 ± 0.55 points, followed by patients and caregivers at 4.33 ± 0.58, nursing work at 4.27 ± 0.51 points, work schedule at 4.23 ± 0.60 points, interpersonal relationships at 4.18 ± 0.56 points, and professional knowledge at 4.15 ± 0.61 points. The mean score for turnover intention was 3.48 ± 0.72 out of five points, and the mean score for mindfulness was 3.46 ± 0.64 out of five points. In the sub-domains of mindfulness, awareness of present time had the highest score at 3.72 ± 0.66 points. The mean score for quality of work life was 3.81 ± 0.53 out of six points. In detail, among the sub-domains, hospital welfare policies showed the highest score at 4.80 ± 0.78 points, followed by communication and teamwork at 4.02 ± 0.77 points, hospital management at 3.41 ± 0.83 points, work design for quality nursing at 3.37 ± 0.75 points, and working conditions at 3.02 ± 0.39 points (Table 2).

### 3.3. Differences in Quality of Work Life According to Demographic Features

Differences in the quality of work life were analyzed according to general characteristics. The quality of work life significantly differed according to total household income (F = 2.97, *p* = 0.020), current position (F = 6.64, *p* = 0.002), work satisfaction (F = 31.18, *p* < 0.001), and subjective health (F = 9.78, *p* < 0.001). Post-hoc analysis showed that quality of work life score increased in those with a total household income of >USD 4200 compared to those with a total household income < USD 3300. Additionally, the quality of work life was higher in head nurses than in general nurses. Those who were greatly and moderately satisfied with their work showed a greater quality of work life score than those who were moderately satisfied and unsatisfied with their work, respectively. Participants with good subjective health also had a greater quality of work life than those who had moderate and bad subjective health (Table 3).

### 3.4. Correlations between Job Stress, Turnover Intention, Mindfulness, and Quality of Work Life

Quality of work life was negatively correlated with job stress (r = −0.36, *p* < 0.001) and turnover intention (r = −0.45, *p* < 0.001) and positively correlated with mindfulness (r = 0.35, *p* < 0.001). Moreover, quality of work life was negatively correlated with all sub-domains of job stress. Quality of work life showed the strongest correlation with patients and caregivers (r = −0.32, *p* < 0.001), followed by role conflicts (r = −0.30, *p* < 0.001), nurse compensation (r = −0.29, *p* < 0.001), nursing work (r = −0.28, *p* < 0.001), interpersonal relationship (r = −0.26, *p* < 0.001), work schedule (r = −0.26, *p* < 0.001), psychological burden (r = −0.23, *p* < 0.001), professional knowledge (r = −0.18, *p* = 0.010), and conflicts with physicians (r = −0.14, *p* = 0.040) (Table 4).

### 3.5. Factors Affecting Quality of Work Life

To identify the factors affecting quality of work life, a regression analysis was conducted. Household income, current position, work satisfaction, and subjective health were among the general characteristics, which showed significant differences and work stress, turnover intention, and mindfulness. Among the general characteristics of participants, total household income, current position, work satisfaction, and subjective health were treated as dummy variables (Table 5).

Regression analysis showed that the Durbin–Watson value was approximately 2, suggesting that there was no autocorrelation between the error terms. Additionally, all variance expansion factors were less than 10, showing no multi-collinearity problems between the independent variables. Thus, the basic assumptions for multiple regression analysis were satisfied.

Model 1 evaluated the effects of general characteristics on quality of work life. Work satisfaction was a significant influencing factor. Those who were greatly and moderately satisfied with work had a higher quality of work life than those who were moderately satisfied (β = 0.48, *p* < 0.001) and unsatisfied with work (β = 0.29, *p* < 0.001), respectively. Work satisfaction had an explanatory power of 24.0% (F = 7.52, *p* < 0.001) for quality of work life.

In model 2, work stress, turnover intention, and mindfulness were included, in addition to the general characteristics. As a result, work satisfaction, job stress, and turnover intention, were significant influencing factors. Those who were satisfied with work, had lower job stress, and had lower turnover intentions showed a higher quality of work life than those who were not satisfied with work (β = 0.27, *p* = 0.004), had high job stress (β = −0.23, *p* < 0.001), and had high turnover intentions (β = −0.18, *p* = 0.016), respectively. These variables had an explanatory of 33.3% (F = 8.90, *p* < 0.001) for quality of work life, which was 9.3% greater than that observed in model 1.

## 4. Discussion

This descriptive study was conducted to identify the factors affecting quality of work life in nurses working in tertiary general hospitals.

Our findings showed that the mean score for job stress was 4.17 out of five points. Although the tools used to evaluate job stress were different, the score observed in our study was higher than the 2.97 (out of 5) [3] that was evaluated during the COVID-19 pandemic and the 3.01 (out of 5) measured before the pandemic [14] in nurses working in public health centers. Additionally, in China, nurses had a mean score of 3.3 (out of 5) for job stress during the COVID-19 pandemic [39]. These differences in the job stress score may be attributed to the participant group and data collection period. The participants in this study were nurses working in two tertiary general hospitals. A tertiary hospital in Korea aims to provide high-quality medical services for serious diseases and efficiently utilize medical resources through a medical delivery system [40]. This leads to increased stress for nurses. In particular, before the COVID-19 pandemic, job stress in nurses working in tertiary general hospitals was high, at 3.70 points [41]. Moreover, it is thought that the increased amount of work during the pandemic, and the resulting fatigue, burnout, and anxiety contributed to higher levels of job stress. Therefore, a prompt intervention and management would be necessary to relieve job stress in nurses working in tertiary general hospitals.

In this study, the mean score for quality of work life in nurses was 3.81 out of six points. Consistent with our finding, the score for quality of work life was 3.69 in a study of Korean nurses [42] and 3.92 in another study of Taiwanese nurses [26]. Furthermore, we observed that quality of work life was significantly higher in head nurses and increased as household income increased. In agreement with our results, a previous study of nurses working at tertiary general hospitals in Saudi Arabia [22] showed that quality of work life was related to the number of years worked and salary. More clinical experiences in nurses leads to a higher sense of security at work, and this can be related to quality of work life. Therefore, for nurses with relatively less clinical experience, hospitals would need to establish strategies to improve the sense of stability and subsequent quality of work life.

Quality of work life was negatively correlated with job stress and turnover intention in this study. This correlation between quality of work life, job stress, and turnover intention has already been demonstrated in several previous studies [16,17,22]. In particular, our findings showed that, among the sub-domains of job stress, patients and caregivers, followed by role conflict, nurse compensation, and nursing work, had the strongest correlation with quality of work life. These findings may be attributed to the fact that the nurses in our study worked at tertiary hospitals and that data were collected during the COVID-19 pandemic. In other words, confirmed COVID-19 patients and their caregivers, as well as nurses, were highly anxious about the COVID-19 pandemic. The increased quarantine measures led to an increased workload for nurses, and the lack of compensation led to high job stress and, therefore, poor quality of work life. In preparation for possible outbreaks of other infectious diseases after COVID-19, response policies, a division of roles and assignments, and improvements in compensation must be re-evaluated during the COVID-19 pandemic.

Upon analysis, we observed that job stress, work satisfaction, and turnover intention affected quality of work life. Consistent with our findings, in a study evaluating the quality of life and work–life balance in nurses in Singapore [43], the factors affecting quality of work life included ability to cope with stress and work satisfaction. In addition, turnover intention also affected the quality of work life in another study [26], further supporting our findings. Based on these results, hospitals and nursing department managers would need to establish plans to reduce job stress, increase work satisfaction, lower turnover intention, and thereby improve the work-life quality for nurses. In this study, mindfulness was not a factor affecting quality of work life, despite the correlations. These results imply that quality of work life is influenced by factors related to nurses’ tasks, such as job stress and work satisfaction, rather than the individual characteristics of nurses. Herein, data were collected during the worldwide COVID-19 pandemic, and this would have heavily affected the results. However, a previous integrative review of the literature on quality of work life in nurses [44] showed that demographic characteristics, such as age, marital status, and education level, were predicting factors for quality of work life. Thus, further studies should investigate the effects of individual characteristics and emotional state on the quality of work life in nurses.

A few limitations must be considered in the interpretation of this study’s findings. First, in this study, female nurses working in tertiary general hospitals were selected for the survey. In the future, it is necessary to understand the quality of life of all nurses through a study on the quality of work life for male nurses. Second, the organizational culture of the hospital and support from managers, which may be related to quality of work life, were not considered. This may have affected the outcomes of this study. Hence, follow-up studies need to evaluate the organizational culture and managerial characteristics of hospitals and understand the differences in quality of work life according to these characteristics. Third, the cross-sectional design of this study was limited in assessing the causality between the study variables. The COVID-19 pandemic has further highlighted the problems with the nursing community. This study presented basic data to find risk factors and solutions in nurses’ work environment by evaluating the quality of nurses’ work life and, through this, it can be expected to develop a model that can understand the quality of work life and contribute to the development of interventions. These are the important practical and theoretical implications of this study. In addition, since it is the basic job of a nurse to provide face-to-face nursing care, a nurse’s quality of work life is related to the nurse’s job. Interventions regarding the factors affecting quality of work life that were identified in this study will enable quality patient care. 

## 5. Conclusions

This study results showed that nurses had high levels of job stress and moderate quality of work life. Quality of work life showed significant differences according to household income, current position, and work satisfaction. Quality of work life was negatively correlated with job stress and turnover intention, and positively correlated with mindfulness. In addition, work satisfaction, job stress, and turnover intention significantly affected quality of work life. Quality of work life in nurses was affected by factors that the nurses experienced during their work. Although both positive and negative factors had effects on the quality of work life, positive factors such as work satisfaction had stronger effects than negative factors. Thus, it would be necessary to seek strategies such as improving compensation for nurses, enhancing teamwork, or establishing a support system for managers, superiors, and colleagues.

## Figures and Tables

**Table 1:** Demographic features of the participants (*n* = 207).

Characters	Categories	Mean ± SD	*n*	%
Age (year)		36.11 ± 9.16		
Marriage	Single		98	47.3
	Married		107	52.7
Education	Associate degree		33	15.9
	Bachelor’s degree		139	67.1
	Master or above		35	16.9
Total household income *	Less than 2500		32	15.5
	2500–less than 3300		62	30.0
	3300–less than 4100		16	7.7
	4100–less than 5000		24	11.6
	Over 5000		73	35.3
Current position	Staff nurse		159	76.8
	Charge nurse		29	14.0
	Head nurse		19	9.2
Working pattern	Shift		101	48.8
	Full-time		106	51.2
Current department	General ward		68	32.9
	Intensive care unit		22	10.6
	Outpatient department		61	29.5
	Special department		27	13.0
	Others		29	14.0
Total period of clinical experience (Month)		157.34 ± 111.34		
Total period of current department (Month)		54.73 ± 60.49		
Satisfaction with current work	Good		50	24.2
	Moderate		98	47.3
	Bad		59	28.5
Subjective health status	Good		54	26.1
	Moderate		73	35.3
	Bad		80	38.6
Eating habits	Regular		80	38.6
	Irregular		127	61.4
Regular exercise	Yes		47	22.7
	No		160	77.3

* USD.

**Table 2:** Job stress, turnover intention, mindfulness, quality of work life (*n* = 207).

Variables	Min	Max	Mean	SD
Job stress	2.86	5.00	4.17	0.42
Nursing work	2.33	5.00	4.27	0.51
Role conflict	2.20	5.00	4.07	0.60
Professional knowledge	2.00	5.00	4.15	0.61
Conflict with physicians	2.33	5.00	4.54	0.55
Psychological burden	1.67	5.00	4.07	0.65
Interpersonal relationships	2.67	5.00	4.18	0.56
Nurse compensation	1.60	5.00	3.79	0.72
Work schedule	2.14	5.00	4.23	0.60
Patients and caregivers	1.00	5.00	4.33	0.58
Turnover intention	1.67	5.00	3.48	0.72
Mindfulness	1.70	5.00	3.46	0.64
Awareness of present time	1.80	5.00	3.72	0.66
Attention	1.60	5.00	3.53	0.71
Non-judgmental acceptance	1.00	5.00	3.56	0.78
Decentralized attention	1.00	5.00	3.02	0.88
Quality of work life	2.29	5.54	3.81	0.53
Hospital management	1.33	6.00	3.41	0.83
Communication and teamwork	1.86	6.00	4.02	0.77
Hospital welfare policies	2.43	6.00	4.80	0.78
Work design for quality nursing	1.30	5.10	3.37	0.75
Working conditions	1.80	4.00	3.02	0.39

**Table 3:** Differences in quality of work life according to demographic features (*n* = 207).

Characters	Categories	Mean ± SD	t or F	*p* Tukey
Marriage	Single	3.75 ± 0.56	−1.59	0.114
	Married	3.87 ± 0.50		
Education	Associate degree	3.87 ± 0.56	2.48	0.086
	Bachelor’s degree	3.76 ± 0.52		
	Master or above	3.97 ± 0.52		
Total household income *	Less than 2500 ^a^	3.63 ± 0.50	2.97	0.020
	2500–less than 3300 ^b^	3.71 ± 0.57		d, e > a, b
	3300–less than 4100 ^c^	3.90 ± 0.43		
	4100–less than 5000 ^d^	3.98 ± 0.49		
	Over 5000 ^e^	3.91 ± 0.52		
Current position	Staff nurse ^a^	3.75 ± 0.51	6.64	0.002
	Charge nurse ^b^	3.95 ± 0.53		c > a
	Head nurse ^c^	4.17 ± 0.54		
Working pattern	Shift	3.85 ± 0.53	0.85	0.398
	Full-time	3.78 ± 0.54		
Current department	General ward	3.83 ± 0.54	1.25	0.289
	Intensive care unit	4.03 ± 0.53		
	Outpatient department	3.75 ± 0.49		
	Special department	3.80 ± 0.50		
	Others	3.78 ± 0.63		
Satisfaction with job	Good ^a^	4.18 ± 0.43	31.18	<0.001
	Moderate ^b^	3.83 ± 0.51		a > b > c
	Bad ^c^	3.47 ± 0.43		
Subjective health status	Good ^a^	4.01 ± 0.57	9.78	<0.001
	Moderate ^b^	3.81 ± 0.49		a > b, c
	Bad ^c^	3.65 ± 0.49		
Eating habits	Regular	3.86 ± 0.53	0.98	0.326
	Irregular	3.78 ± 0.53		
Regular exercise	Yes	3.94 ± 0.59	1.92	0.057
	No	3.78 ± 0.51		

* USD. a, b, c, d, e subgroups classified based on the mean values of quality of work life.

**Table 4:** Correlation among job stress, turnover intention, mindfulness, quality of work life.

Variables	Job Stress Total	1	2	3	4	5	6	7	8	9	Turnover Intention	Mindfulness
	r *p*											
Job stress total	1											
1. Nursing work	0.68 <0.001	1										
2. Role conflict	0.78 <0.001	0.55 <0.001	1									
3. Professional knowledge	0.72 <0.001	0.37 <0.001	0.67 <0.001	1								
4. Conflict with physicians	0.72 <0.001	0.41 <0.001	0.59 <0.001	0.55 <0.001	1							
5. Psychological burden	0.73 <0.001	0.41 <0.001	0.53 <0.001	0.67 <0.001	0.49 <0.001	1						
6. Interpersonal relationship	0.78 <0.001	0.40 <0.001	0.59 <0.001	0.61 <0.001	0.56 <0.001	0.62 <0.001	1					
7. Nurse compensation	0.74 <0.001	0.34 <0.001	0.46 <0.001	0.44 <0.001	0.45 <0.001	0.50 <0.001	0.55 <0.001	1				
8. Work schedule	0.62 <0.001	0.46 <0.001	0.29 <0.001	0.19 0.0063	0.30 <0.001	0.25 <0.001	0.29 <0.001	0.41 <0.001	1			
9. Patients and caregivers	0.70 <0.001	0.36 <0.001	0.48 <0.001	0.42 <0.001	0.55 <0.001	0.48 <0.001	0.52 <0.001	0.47 <0.001	0.40 <0.001	1		
Turnover intention	0.19 0.006	0.26 <0.001	0.15 0.035	−0.05 0.484	0.09 0.215	−0.00 0.988	0.07 0.340	0.15 0.030	0.24 <0.001	0.18 0.011	1	
Mindfulness	−0.40 <0.001	−0.41 <0.001	−0.37 <0.001	−0.32 <0.001	−0.21 0.003	−0.28 <0.001	−0.28 <0.001	−0.27 <0.001	−0.20 0.003	−0.022 0.002	−0.24 0.001	1
Quality of work life	−0.36 <0.001	−0.28 <0.001	−0.30 <0.001	−0.18 0.010	−0.14 0.040	−0.23 0.001	−0.26 <0.001	−0.29 <0.001	−0.26 <0.001	−0.32 <0.001	−0.45 <0.001	0.35 <0.001

**Table 5:** Factors affecting quality of work life (*n* = 207).

		Model 1			Model 2		
		β	t	*p*	β	t	*p*
	(Constant)		34.60	<0.001		10.11	<0.001
Demographic characteristics	Monthly income * (=Less than 2500)	−0.04	−0.50	0.618	−0.09	−1.27	0.205
	Monthly income * (=2500–less than 3300)	−0.10	−1.26	0.210	−0.12	−1.64	0.103
	Monthly income * (=3300–less than 4100)	0.01	0.10	0.917	−0.04	−0.58	0.566
	Monthly income * (=4100–less than 5000)	0.05	0.75	0.456	0.00	0.02	0.988
	Position (=charge nurse)	0.05	0.75	0.455	0.05	0.80	0.426
	Position (=head nurse)	0.10	1.46	0.146	0.07	1.08	0.284
	Satisfaction with job (=Good)	0.48	5.86	<0.001	0.27	2.91	0.004
	Satisfaction with job (=Fair)	0.29	3.69	<0.001	0.12	1.48	0.140
	Subjective health status (=Good)	0.07	1.01	0.313	0.03	0.41	0.680
	Subjective health status (=Bad)	0.05	−0.67	0.503	−0.00	−0.02	0.983
Job stress					−0.23	−3.57	<0.001
Turnover intention					−0.18	−2.43	0.016
Mindfulness					0.12	1.85	0.066
		Adjusted R^2^ = 0.240 F = 7.52, *p* < 0.001			Adjusted R^2^ = 0.333 F = 8.90, *p* < 0.001		

* USD.

## Data Availability

The data presented in this study are available on request from the corresponding author. The data are not publicly available due to participants’ privacy.
